# Relationship between Macrovascular and Microvascular Hemodynamics Assessed by Spectrophotometry in Periodontal Diseases

**DOI:** 10.1155/2021/9925198

**Published:** 2021-06-03

**Authors:** M. M. Figliuzzi, S. Sestito, D. Pacifico, L. Parentela, Carlo Rengo

**Affiliations:** ^1^Medicine and Surgery School, Department of Health and Oral Sciences Periodontology Clinic, “Magna Graecia” University of Catanzaro, Catanzaro, Italy; ^2^University of Naples “Federico II”, Napoli, Italy

## Abstract

**Objective:**

The aim of this study is to identify a possible link between macrovascular hemodynamic status and microvascular hemodynamic indices in patients with periodontal disease.

**Methods and Materials:**

Seventeen adult patients are recruited on a voluntary basis at the Dentistry Department of the “Mater Domini” University of Catanzaro, with sampling that determines the lipid profile, blood glucose, inflammatory mediators, blood plasma viscosity: anamnesis, blood pressure measurement, and detection of anthropometric parameters: eco-Doppler of the carotid arteries and brachial arteries with noninvasive measurements of hemodynamics and evaluation of inflammation and periodontal circulation with a noninvasive spectroscopic technique. The subjects underwent a dental inspection with periodontal proves. The different indices of periodontal disease were evaluated.

**Results:**

The sites with high probing depth differ from the healthy ones, showing low oxygen saturation and a notable increase in tissue edema, but no correlation between macro- and microvascular values was found.

**Conclusion:**

Periodontal probing and spectroscopic examination showed the correlation between low oxygen saturation levels and tissue edema values with probing depth; however, no correlation between macrovascular hemodynamic status and microvascular hemodynamics indices was found probably given the heterogeneity of the population under consideration, the low number of data gathered, and the small sample size.

## 1. Introduction

The association between dental and cardiovascular diseases has been arousing much interest in the recent years.

Some authors have investigated the correlation between the levels of some chronic inflammation mediators present in the blood and saliva and the severity of periodontal disease.

Recently a study wanted to analyze and identify the association among salivary interleukin-6 (IL-6) levels and periodontitis (PT) and to determine the significant trend of this association in PT patients.

Patients in the PT group had significantly higher median salivary IL-6 levels compared to the HS group. Salivary IL-6 levels were negatively correlated with C-reactive protein, with the number of teeth and with clinical attachment loss (CAL), probing pocket depth (PPD), and bleeding sites (FMBS). Furthermore, in patients with PT, salivary IL-6 levels were inversely associated (P-trend) with the number of teeth, and directly associated with the proportional extent of PT; these results so showed that PT patients presented significant higher salivary IL-6 levels compared to HS. Moreover, in the analyzed sample, a significant p-trend among PT, tooth loss, and increased salivary IL-6 levels was found [1].

Another study from the same group of authors aimed to analyze the association and impact of periodontitis and coronary heart disease (CHD) on salivary and serum galectin-3 in patients with periodontitis and CHD; the results showed that patients with periodontitis and periodontitis + CHD presented significant higher serum and salivary galectin-3 levels in comparison with CHD patients and healthy subjects. Periodontitis and ET-1 were the significant predictors of serum and salivary galectin-3 levels, respectively. [2].

Another study found increased leukotriene concentration in the gingival crevicular fluid from subjects with periodontal disease and atherosclerosis. [3].

Various studies have reported that there is a correlation between CHD risk factors or metabolic syndrome and related diseases (arterial hypertension, obesity, or diabetes) and periodontal disease, and this is very interesting due to the potential clinical implications [4, 5].

Hemodynamics is that branch of cardiovascular physiology that analyzes and studies the behavior of blood moving in the vessels, therefore allowing reducing the danger of forming pathologies related to blood circulation.

The functioning of the cardiocirculatory system can be explained using the physical principles derived from the laws of hydrostatics-hydrodynamics, although the cardiocirculatory system has features that prevent a precise quantitative description.

In fact, the cardiocirculatory system presents

- elastic and nonrigid ducts
- tracts (capillaries) that allow the leakage and the entry of liquids
- a pump (heart) with intermittent activity
- external pressure in the ducts which can determine changes in the caliber due to its elasticity
- a non-Newtonian fluid in motion (blood), with viscosity varying with variation of speed

The blood is a complex fluid consisting of a suspension of particles in liquid form (plasma) and, as such, subject to variations in viscosity. The blood viscosity depends on the temperature and hematocrit. Usually, we consider the blood viscosity to be around 4·103 kg m^−1^ s^−1^ at a temperature of 37°C and a hematocrit of 45. Basically, the viscosity can be considered as a resistance in flow, given that the layers of fluid subjected to intermolecular attractions oppose a deformation.

The monitoring of hemodynamic forces in patients with periodontal disease could be important for an accurate evaluation of the overall cardiovascular risk for periodontal therapy [6].

The wall shear stress is the frictional force that is generated by the blood when flowing tangentially to the endothelial surface, strongly influencing the arterial remodeling and, if low, promotes the formation of plaques.

It is known that the wall shear stress is lower in elder people and in those with high blood pressure, high body mass index, or affected by diabetes [7, 8]. Moreover, the wall shear stress is generally different between males and females as the periodontal disease, tooth loss, and atheroschlerosis [9]. Furthermore, this association is independent of the classic CHD risk factors and age. It has been proved that the wall shear stress decreases with the worsening of periodontal health, potentially causing atherogenic local flow conditions.

The way in which periodontitis affects hemodynamics could be the systematic inflammation [10], with an enlargement in arterial diameter causing, for example, rheumatoid arthritis. Several studies have proven that inflammatory mediators such as interleukins and TNF-*α*, produced by the inflamed oral tissues [11], can activate systemic inflammation and the subsequent immune cascade, with the resulting low-grade chronic inflammation which should promote the development of atherosclerotic disease [12]. Furthermore, a low shear stress could in turn increase the local vascular inflammation.

The calculation of the hemodynamic forces is based on the eco-Doppler measurements: this method is fast, repeatable, of low cost, valid, and vastly applied in this field.

The aspect investigated in this study is the association between local carotid hemodynamics, microvascular hemodynamics indices, and periodontal disease, and the purpose is to identify the link between periodontal indices and micro- and macrovascular hemodynamics values, in particular the wall shear stress, suggesting that the alternation of the hemodynamic profile could contribute to atherosclerosis in subjects with periodontal disease.

## 2. Methods and Materials

Seventeen adult patients who meet the inclusion criteria are sequentially recruited on a voluntary basis at the dentistry crime of the “Mater Domin” University Hospital of Catanzaro; those who meet the inclusion criteria signed written consent.

### 2.1. The Inclusion Criteria

- Patients with periodontal disease requiring dental therapy (moderate or advanced periodontal lacerations)

### 2.2. The Exclusion Criteria

- Atrial fibrillation
- Significant hemodynamically carotid stenosis
- Use of drugs with an antihypertensive effect
- Nonmenopausal women or neoplasm in act

Informative consent is signed by each subject before the spectra are collected. The periodontal sites are defined as those with periodontal probing depth ≥5 mm and a loss in clinical attachment of ≥3 mm. Gingivitis sites are defined as those with periodontal probing depth <3 mm and bleeding on probing. Healthy sites are defined as those with probing depth <3 mm and no bleeding on probing. The spectra were obtained from the contralateral healthy sites or the closest contralateral site closest to the diseased site. The spectra measurements were acquired in the following site: Mea-Buccal, Mid-Lingual, Mesio-Buccal, Mesio-ingual, Disto-Buccal and Disto-Lingual.

The observational study clinic is based on the addition of a cardiovascular screening along with macro- and microvascular hemodynamic measures of selected dental patients. It is made up of

- blood sampling in order to determine the lipid profile, glycemia, inflammatory mediators, and blood and plasma viscosity
- anamnesis, blood pressure measurement, and detection of anthropometric parameters
- eco-Doppler ultrasound of the brachial carotid arteries with noninvasive measurement of hemodynamics
- evaluation of the inflammation and periodontal circulation with a noninvasive spectroscopic technique

Systolic and diastolic blood pressures were measured by using a standardized sphygmomanometer on the right arm of the participants, after having rested for at least 5 minutes. An average of the second and third reading is then calculated.

The observational clinic study is, therefore, based on the addition of cardiovascular screening and of macro- and microvascular hemodynamic measures towards selected dental patients. It consists of blood sampling in order to determine the lipid profile, glycemia, and inflammatory mediators with blood and plasma viscosity; anamnesis, blood measurement, and detection of anthropometric parameters; Doppler ultrasound of the carotid and brachial arteries with noninvasive measurements of hemodynamics; and evaluation of inflammation and periodontal circulation with a noninvasive spectroscopic technique. Weight and height are measured with routine methods. The body mass index (BMI) was calculated as weight (kg) divided by the height squared. Patients with values higher than 29.9 kg7 m2 were considered to be obese. Glucose and lipids present in the blood were measured with kits available on the market. The eco-Doppler exam was conducted with an ATL 3000 activated ECG instrument equipped with a 5–0 MHz multifrequency linear probe.

The common carotid artery, the carotid bulb, and the internal and external carotid artery have all been studied longitudinally and transversally, with anterior, lateral, and posterior approaches. Each segment has been classified as normal, with plaque, and with stenosis. A segment with plaque showed a localized laceration which invaded the lumen by >1,3 mm, without enlarging the spectral or only in the deceleration phase of the systole, with systolic and peak flow velocity of <120 cm/s. The segments with stenosis showed a spectral enlargement during the systole or a peak flow in systolic velocity of <120 cm/s (Table 1).

The peak and mean of the wall shear stress were measured in the common carotid 1-2 cm next to the bulb with the following formulas:

- Peak: 4*η*VSP\IDT
- Average: 4*η*VM\IDR

Subjects underwent dental inspection with periodontal probes.

- The gingival plaque was calculated using the Silness and Loe plaque index, while the gingival inflammation was assessed using the gingival index.
- The depth of the pocket is evaluated. The gingival margin was taken as reference point when reading the values during probing.

As it is known, the periodontal indices are usually a sum of each dental element score, divided by the number of teeth, resulting in an average value for the patient. In this work, the sum of the values was calculated for each periodontal index of each patient. The staff performing the tests was unaware of the other measurements.

## 3. Results

The 17 patients in this preliminary phase presented an age range with varied from 23 to 74 years with an average of 49.8 years. The group was made up of 9 males and 8 females. Looking at the comparison between the periodontal probing and the spectroscopic examination, as already occurred in previous works of Liu [13, 14], the correlation between low oxygen saturation levels and the tissue edema values with the depth of the pol was confirmed. In fact the sites with gingivitis and the healthy sites presented superimposable spectra, while on the other hand, sites that presented an extensive depth of probing differed greatly from the healthy sites, highlighting the low oxygen saturation and a notable increase in the tissue edema. The hemodynamic and clinical results are presented in Table 2.

The variability in measurements of macro- and microvascular hemodynamics was not correlated in the population under examination (*p* > 0.1).

The statistical analysis applied was a multiple linear correlational analysis made with SPSS v.17.0.

## 4. Discussion

The comparison between periodontal probing and spectroscopic examination showed, as in the past, the correlation between low oxygen saturation levels and tissue edema values with probing depth; in fact, the sites with high probing probability differ from the healthy ones, showing a low oxygen saturation and a notable increase in tissue edema. However, no correlation between macrovascular haemodynamic status and microvascular haemodynamic indices was found in the small population under examination. The negative result of the study could be attributed to numerous reasons. The most likely hypothesis is that, also given the heterogeneity of the population under consideration, the low number of data gathered affected the result. The statistic test used should correct these factors but probably not with great effectiveness given the small sample size. The other hypothesis is that the spectrophotometric measurements, indirect for hemodynamics, were represented very little by the gingival microcirculation for there to be a strong and, therefore, visible association with the already few macrovascular hemodynamics. Finally, it is possible that the two circulatory districts were not strongly connected in the gingival pathology, and due to the enlargement of the local gingivopathy, it was only the systematic inflammation which gave the verified worsening of the carotid hemodynamic situation in patients with periodontal disease.

## Figures and Tables

**Table 1:** Parameters used in formulas.

ID	Internal diameter; distance from the near and distant lumen-intimate interface measured with the echography
IDR	Measured at time *R* of the ECG
IDT	Measured at time *T* of the ECG
VSP	Peak systolic velocity
VM	Mean systolic velocity

**Table 2:** Hemodynamic and clinical results.

Parameters	Average or percentage	Standard deviation
Age (years)	49.8	15.4
Peak_shear_stress carotid (dynes/cm^2^)	25.3	6.5
Mean shear carotid (dynes cm^2^)	10.2	3.5
Tissue oxygen saturation	27.1	3.4
Tissue water content	11.3	1.1
Obesity (%)	18	
Dislipidemia (%)	12	
Hyertention (%)	18	
Diabetes (%)	0	
Smoke (%)	35	
Alcohol (%)	18	
Cardiovascular diseases (%)	6	
Pathological carotid eco-Doppler (%)	6	

## Data Availability

The data are private as they are of patients of the Mater Domini University Hospital; they can be obtained on request from the corresponding author.
